# Clinical and psychosocial remission in schizophrenia: correlations with antipsychotic treatment

**DOI:** 10.1186/1471-244X-12-108

**Published:** 2012-08-10

**Authors:** Yoram Barak, Dov Aizenberg

**Affiliations:** 1 Psychiatry Department, Sackler School of Medicine, Tel-Aviv University, 12 H Levanon Street, Tel-Aviv, 69978, Israel; 2 Department E, Geha Mental Health Center, 1 Helsinki Street, Tel-Aviv, 49100, Israel; 3 Psychogeriatric Department, Abarbanel Mental Health Center, 15 KKL Street, Bat-Yam, 59100, Israel

**Keywords:** Schizophrenia, Remission, Symptomatic, Social

## Abstract

**Background:**

Clinical and psychosocial remission amongst persons with schizophrenia is nowadays a defined goal of treatment. This necessitates incorporating quantifiable psychosocial variables with traditional symptomatic data. We aimed to assess clinical and psychosocial remission in schizophrenia in a large cohort of community dwelling persons with schizophrenia. We emphasized between-groups comparison of antipsychotic medications and administration methods on the outcome of remission.

**Methods:**

Psychiatric case managers rated psychosocial remission using the PsychoSocial Remission Scale (PSRS) and clinical remission using the Remission in Schizophrenia Working Group symptomatic remission criteria (RSWG). Ratings were performed for persons with schizophrenia they have been treating for 6 months or more. Data as to gender, age and pharmacological treatment of each patient were also collected.

**Results:**

Of 445 participants who completed the survey, 268 (60%) were evaluated by psychiatrists, 161 (36%) by nurses and 16 (4%) were evaluated by social workers. Patients mean age was 43.4 + 13.1 years; 61% were men and 39% were women. Antipsychotic treatments were as follows: Per-os (PO) 243 (55%), IM long-acting typical antipsychotics (LAT) 102 (23%) and IM long-acting risperidone (RLAI; Consta) 100 (22%). Overall, 37% of patients achieved symptomatic remission and 31% achieved psychosocial remission. Rates of symptomatic remission were significantly higher in patients treated by LAT and RLAI compared with PO (51% and 48% vs., 29% respectively, p = 0.0003). Rates of psychosocial remission were also significantly higher in patients treated by LAT and RLAI compared with PO (43%% and 41% vs., 24% respectively, p = 0.003).

**Conclusion:**

In a large national sample a third of persons with schizophrenia were in remission. IM long acting preparations were associated with higher remission rates. Treatment choice may thus influence rates of remission in persons with schizophrenia.

## Background

In recent years there has been an emphasis on meaningful clinical outcomes as well as focus on functional recovery in mental health. In schizophrenia, complete recovery implies the ability to function in the community, socially and vocationally, as well as being relatively free of disease-related symptomatology [1]. A consensus proposal by Andreasen et al. [2] presented criteria to define symptomatic remission in schizophrenia. The group proposed that symptom remission be based on maintenance of low level of symptoms for at least 6 months in psychoticism, disorganization, and negative symptoms. Since the publication of the Remission in Schizophrenia Working Group symptomatic remission criteria (RSWG) scale in 2005 many studies have employed this instrument to assess the characteristics and epidemiology of symptomatic remission amongst persons with schizophrenia. The results of these studies emphasize that the remission concept appears to be achievable and is not just an academic definition. More than a third of patients being treated with antipsychotics meet the full symptomatic remission criteria [3].

Consensus-defined standards for symptomatic remission in persons with schizophrenia have been the focus of clinical and research interest in the last decade [2]. Such standards provide clarity for treatment goals, as well as a framework for the design and comparison of investigational trials and the assessment of the effectiveness of pharmacological and psychosocial interventions.

It is accepted that impairments in everyday living skills are present in persons with schizophrenia and the current diagnostic criteria of schizophrenia includes several references to functioning. The alleviation of schizophrenia symptoms, which has clearly improved with the development of antipsychotic medications with greater efficacy, does not necessarily mirror a similar alleviation of functional impairments. Symptomatic and psychosocial remissions appear to be different domains, representing two steps towards recovery that do not necessarily overlap [4]. Persons with schizophrenia are capable of achieving symptomatic remission and high levels of social functioning [1]. Psychosocial function and dysfunction are of critical importance in achieving remission yet there is still a scarcity of studies addressing this issue using formal quantifiable rating scales.

We have recently developed the Psychosocial Remission in Schizophrenia (PSRS) Scale - an 8-item scale quantifying psychosocial remission in schizophrenia [5]. The PSRS is a user-friendly clinician rated scale for the assessment of psychosocial remission in schizophrenia that complements the Remission in Schizophrenia Working Group scale [2]. Psychiatric case managers rated psychosocial remission using the PSRS and clinical remission using the Remission in Schizophrenia Working Group symptomatic remission criteria (RSWG). The aim of the present study was to quantify both symptomatic and psychosocial remission in a national sample of community dwelling persons with schizophrenia using these validated and accepted rating scales. The study reflects between-groups comparison of antipsychotic medications and oral versus depot long acting preparations administration methods on the outcome of remission.

## Method

The 2005 APA Remission in Schizophrenia Working Group (RSWG) concluded that any definition of remission in schizophrenia should include a significant time component and be applicable to patients across stages of disease course. A symptom-based, validated assessment instrument was developed to enable clinicians and researchers to define symptomatic remission throughout the course of illness [2]. In brief, this instrument proposed using 8 items from either (a) the PANSS; or (b) the Scale for Assessment of Positive Symptoms (SAPS) and Scale for Negative Symptoms (SANS); or (c) the Brief Psychiatric Rating Scale (BPRS). These proposed criteria were put forward to ensure that three dimensions of psychopathology are to be assessed – Psychoticism, Disorganization and Negative symptoms [6]. A patient suffering from schizophrenia is defined as being in remission if for the past 6 months he/she scored mild/minimal/absent on each of the 8 items of the symptomatic remission scale (RSWG).

The Israel Psychiatric Association has decided to adopt this approach. A task force was formed to develop the PSRS and was instructed that it should create a tool to complement the RSWG so that it too include 8 items, each scored from 1 – absent, to: 7 – extreme. A questions’ bank was reviewed by 429 mental health professionals – psychiatrists, residents, psychiatric nurses, and community nurses. Four items were found to be most frequently sanctioned in the quality-of-life domain (familial relations, understanding and self-awareness, energy and interest in everyday life). Four items were sanctioned in the instrumental activities of daily living domain (self-care, activism, responsibility for medications and use of community services). The inter-rater reliability for each of the 8 PSRS items was calculated (interclass correlation) to be as follows: item 1 – 0.83; item 2 – 0.68; item 3 – 0.66; item 4 – 0.70; item 5 – 0.67; item 6 – 0.78; item 7 – 0.76 and item 8 – 0.68. In addition the dichotomous distinction between “in-remission” (scoring 1, 2 or 3 on each item) or “non-remission” (scoring 4 or higher) was computed. The mean frequency of correlated score was 80% ranging from 64% for the “Impaired Activism” item to 90% for the “Impaired Understanding and Self- awareness” item. Interrater reliability among 70 psychiatrists ranged from 0.67 to 0.83. [For detailed description of the PSRS see (Barak et al, 2010; Ref., [5])].

A survey questionnaire was designed consisting of 3 sections: a) clinical and demographic variable; b) the RSWG scale and c) the PSRS (See Table 1). Survey questionnaires were distributed in community mental health centers across the country. All healthcare survey participants were provided with the same instructions to complete the rating of both scales following an interview with the patient. Psychiatric case managers rated psychosocial remission using the PsychoSocial Remission Scale (PSRS) and clinical remission using the Remission in Schizophrenia Working Group symptomatic remission criteria (RSWG). The target population for participation in this survey was composed of persons with schizophrenia fulfilling the following criteria: 1) DSM-IV diagnosis of schizophrenia, 2) age 18 years or older, 3) no psychiatric hospitalization or a minimum of 6 months following discharge from inpatient psychiatric care, 4) a mental health professional case-manager in charge of the patient for a minimum of 6 months and 5) no change in psychotropic medications in the 4 weeks preceding the survey evaluation.

**Table 1:** The PSRS scale

**Severity**							**PSRS items**
1	2	3	4	5	6	7	Impaired Familial Relations
1	2	3	4	5	6	7	Impaired Understanding and Self- awareness
1	2	3	4	5	6	7	Impaired Energy
1	2	3	4	5	6	7	Impaired Interest in daily life
1	2	3	4	5	6	7	Impaired Self-care
1	2	3	4	5	6	7	Impaired Activism
1	2	3	4	5	6	7	Impaired Responsibility Medical Treatment
1	2	3	4	5	6	7	Impaired Use of Community Services

Participants in the present survey were psychiatrists, registered nurses and social workers who were case managers of persons with schizophrenia in community mental health centers across the country.

This study was approved by the Abarbanel MHC Internal Review Board.

### Statistical analysis

Data analyzes was carried out with the use of the Statistical Analysis System software, SAS Institute. The non-parametric sign-rank test method was employed. The t-test and a non-parametric test were undertaken to assess differences between the evaluations for continuous variables (age, scales score). Differences between categorical parameters were tested according to the McNemar test for dependent (matched) populations (gender, case manager profession, dichotomous definition of remission). We employed the non-parametric test as a “conservative” test to ensure analyses in case of skewed data. All tests applied were two-tailed, and p value of 5% or less was considered statistically significant.

## Results

A total of 445 survey questionnaires were endorsed and analyzed in the present study. The majority (268/445; 60%) of case-managers endorsing the questionnaire were psychiatrists. Nurses comprised more than a third of the respondents (161/445; 36%) and social workers only 4% (16/445). The proportion of case managers reflects the employment in Israel within the public mental health sector.

Patients characteristics were as follows: mean age 43.4 + 13.1 years; 61% (N = 271) were men and 39% (N = 174) were women. The most common form of antipsychotic treatment was oral medications (55%; N = 235) followed by Intra Muscular (IM) long acting first generation antipsychotic medications (23%; N = 98) closely followed by IM long acting risperidone (22%; N = 97). There were 15 patients who had been treated by a combination of IM long acting antipsychotic augmented by an oral preparation. These were excluded from the final analysis.

### Symptomatic remission

Of the 445 patients 37% (N = 165) achieved symptomatic remission as assessed by the RSWG scale (mean score 22.8 + 10.6). Rates of symptomatic remission were significantly higher in patients treated by IM long acting first generation antipsychotic medications and IM long acting risperidone compared with oral medications (51% and 48% vs., 29% respectively, p = 0.0003).

### PsychoSocial remission

Of the 445 patients 31% (N = 138) achieved psychosocial remission as assessed by the PSRS scale (mean score 26.3 + 11.8). Rates of symptomatic remission were significantly higher in patients treated by IM long acting first generation antipsychotic medications and IM long acting risperidone compared with oral medications (43% and 41% vs., 24% respectively, p = 0.0003).

No statistically significant differences or trends were observed in either symptomatic or psychosocial remission for analysis by gender, age or by profession of case manager (physician, nurse or social worker).

The two items of the PSRS scale most frequently quantified as denoting remission were “impaired self care” and “impaired responsibility for medical treatment.”

### Non-remitted patients

Although remission was defined dichotomously for the purpose of analyses in the present study, it is important to report how the rest of the sample’s scores were distributed amongst participants. It is of note that women had achieved higher rates of both symptomatic and psychosocial remission than men; 39.5% vs., 36.6% and 34.9% vs., 28.4%, respectively. Post-hoc deletion of the “6 months duration” criteria for remission resulted in lower remission rates for both symptomatic remission (25%) and psychosocial remission (29.5%). Finally, the distribution of remission scores emphasizes that 29.7% of patients were lacking only 2 to 3 points in order to reach the “dichotomous” definition of either symptomatic or psychosocial remission.

### Comparison of remitted and non-remitted patients according to medication

Patients differed in remission status according to antipsychotic medication as follows: a) Oral medications – 51% of remitted patients vs., 49% of non-remitted, b) FGA long acting – 68% of remitted vs., 32% of non-remitted and c) Risperidone long acting – 65% of remitted vs., 35% of non-remitted.

## Discussion

In the present study, a large national survey amongst community dwelling persons with schizophrenia employed two complementary scales to quantify both symptomatic and psychosocial remission rates. The rates of symptomatic remission in the present survey (37%) are in line with recent publications focusing on remission. Haro and colleagues conducted a one - year observational study in Europe following more than 6,500 patients reporting that 38% achieved symptomatic remission [7]. De Hert and colleagues evaluated in a naturalistic prospective study persons with schizophrenia in different treatment settings. In this sample of 341 patients 29% met criteria for symptomatic remission at study endpoint [8]. Recently, Lambert and colleagues aimed to measure symptomatic and functional remission in patients treated with long-acting injectable risperidone. Among 529 patients from seven European countries, symptomatic remission lasting 6 months or longer occurred at some point during treatment in 33% of patients [9]. In the present survey patients who were treated by long-acting injectable antipsychotics – whether first or second generation - were noted to achieve higher rates of symptomatic remission than patients receiving oral antipsychotic treatment. One possible interpretation of these findings is that improved adherence frequently reported with long acting antipsychotics is associated with remission [10]. However, schizophrenia patients with unstable disease may not fully benefit from this advantage of injectable long-acting antipsychotics [11]. Thus, both the fact that patients in the present study had a case manager and the fact that they were not at imminent risk for hospitalization but rather stable at baseline receiving community mental health services, help explain the differences in remission rates between studies.

Evaluating only clinically pathological signs and symptoms does not capture the full range of remission in persons with schizophrenia as social and psychological aspects of their lives may be underrepresented in such evaluations. One of the major aspects of remission is patients’ ability to adapt to the demands of society, but may be harder to achieve than symptomatic stability [12]. Previous social functioning has consistently been found to be a good predictor of future social functioning in schizophrenia. This may be explained by the protective role of social networks [13]. The employment of psychosocial outcomes in studies of schizophrenia patients may elevate the threshold for remission. It has been demonstrated that although psychiatric treatment effectively reduces severity of psychotic symptoms, the majority of patients do not have a satisfactory level of social functioning [14]. Indeed, in the present survey rates of psychosocial remission were lower than for symptomatic remission. This is in accordance with other publications demonstrating that combined symptomatic, functional, and quality-of-life remission was observed only in one in five schizophrenia patients [9].

Contemporary research examines dysfunction among the lives of persons with schizophrenia as a matter of the impact of biological and social forces [15,16]. While literature from a range of sources has explored self-experience in schizophrenia it remains unclear whether these differing views of self-experience are comparable with one another. There is a wide-ranging, if general consensus, which suggests that many persons with schizophrenia experience themselves as diminished relative to their former selves. Within this broad consensus, significant disagreements exist and recent work suggests a program of research to address these disagreements [15]. Recovery is a versatile concept, and the need for its’ quantification in practice has been identified. An analysis of 30 international proposals offering recovery-oriented practice guidance was conducted in 2011. The emerging conceptual framework consists of 16 central themes, grouped into four domains: promoting citizenship, organizational commitment, supporting personally defined recovery, and working relationship. A key challenge for professionals is the lack of clarity about what constitutes recovery-oriented practice [16]. The combination of clinical and psychosocial domains undertaken in the present study may contribute to a better defined recovery.

Recovery is increasingly a focus in mainstream psychiatry. However, it has also become clear that the concept lacks a strong scientific basis. One of the main conceptual issue that has not received sufficient attention in the literature is the role of evidence-based practices in recovery-oriented care [17]. Investigations as to the relation between objective clinical recovery as defined by symptom severity and level of functioning, and subjective personal recovery as defined by quality of life, domains of personal confidence and hope, willingness to ask for help and reliance on others have been sparse. Silverstein & Bellack addressed this issue recently, concluding that that clinical objective recovery is not synonymous with personal subjective recovery yet can be conceptualized as complementary [18]. The present study is in accord with the construct of recovery put forward by these authors as involving both objective and subjective elements [17,18].

Several demographic, clinical, and methodological variables are uncontrolled for in the analyses between drug treatments, limiting the conclusions that can be drawn from this survey. Duration of illness, number of hospitalizations, lifetime exposure to antipsychotic drug dosage and socioeconomic status are unreported. All of these variables, or some combination of these, may have influenced the present study’s results. In addition, it can not be ruled out that regular contact with the case manager may have affected the results instead of the medication itself, differing capacity for metacognition and other variables not captured in this brief survey. Finally, the generalizability of the findings given the restricted sample used must be viewed cautiously.

## Conclusion

In conclusion, the rates of symptomatic remission captured in this national survey are similar to those reported from other countries. To the best of our knowledge this is the first study to report rates of psychosocial remission using a validated rating scale. Psychosocial remission rates were found to be lower than symptomatic remission underlining the need to address this issue through a variety of interventions. As we do not yet have a complete understanding of factors affecting psychosocial remission, adherence may be proposed as a contributing factor. IM long acting preparations in the present survey were associated with higher remission rates, and thus treatment choice may influence rates of remission in schizophrenia patients. It must be stressed that a variety of non-pharmacological interventions as well as a range of empirically validated psychosocial approaches are employed in achieving remission for persons with schizophrenia.

To support these preliminary findings prospective studies quantifying symptomatic and psychosocial remission are called for.

## Competing interests

The authors declare that they have no competing interests.

## Authors’ contributions

YB carried out the preliminary survey and drafted the manuscript. DA carried out the participated in the design of the study. YB conceived of the study, and participated in its design and coordination and helped to draft the manuscript. All authors read and approved the final manuscript.

## Pre-publication history

The pre-publication history for this paper can be accessed here:

http://www.biomedcentral.com/1471-244X/12/108/prepub
